# CIB1 depletion with docetaxel or TRAIL enhances triple-negative breast cancer cell death

**DOI:** 10.1186/s12935-019-0740-2

**Published:** 2019-02-04

**Authors:** Alexander H. Chung, Tina M. Leisner, Gabrielle J. Dardis, Marissa M. Bivins, Alana L. Keller, Leslie V. Parise

**Affiliations:** 1 Department of Pharmacology, University of North Carolina at Chapel Hill, CB #7365, Chapel Hill, NC 27599 USA; 2 Department of Biochemistry and Biophysics, University of North Carolina at Chapel Hill, CB #7260, Chapel Hill, NC 27599 USA; 3 Lineberger Comprehensive Cancer Center, Chapel Hill, NC USA

**Keywords:** CIB1, TRAIL, Apoptosis, Triple-negative breast cancer, Chemoresistance

## Abstract

**Background:**

Patients diagnosed with triple negative breast cancer (TNBC) have limited treatment options and often suffer from resistance and toxicity due to chemotherapy. We previously found that depleting calcium and integrin-binding protein 1 (CIB1) induces cell death selectively in TNBC cells, while sparing normal cells. Therefore, we asked whether CIB1 depletion further enhances tumor-specific killing when combined with either the commonly used chemotherapeutic, docetaxel, or the cell death-inducing ligand, TRAIL.

**Methods:**

We targeted CIB1 by RNA interference in MDA-MB-436, MDA-MB-231, MDA-MB-468, docetaxel-resistant MDA-MB-436 TNBC cells and ME16C normal breast epithelial cells alone or combination with docetaxel or TRAIL. Cell death was quantified via trypan blue exclusion using flow cytometry and cell death mechanisms were analyzed by Western blotting. Cell surface levels of TRAIL receptors were measured by flow cytometry analysis.

**Results:**

CIB1 depletion combined with docetaxel significantly enhanced tumor-specific cell death relative to each treatment alone. The enhanced cell death strongly correlated with caspase-8 activation, a hallmark of death receptor-mediated apoptosis. The death receptor TRAIL-R2 was upregulated in response to CIB1 depletion, which sensitized TNBC cells to the ligand TRAIL, resulting in a synergistic increase in cell death. In addition to death receptor-mediated apoptosis, both combination treatments activated a non-apoptotic mechanism, called paraptosis. Interestingly, these combination treatments also induced nearly complete death of docetaxel-resistant MDA-MB-436 cells, again via apoptosis and paraptosis. In contrast, neither combination treatment induced cell death in normal ME16C cells.

**Conclusion:**

Novel combinations of CIB1 depletion with docetaxel or TRAIL selectively enhance naive and docetaxel-resistant TNBC cell death while sparing normal cell. Therefore, combination therapies that target CIB1 could prove to be a safe and durable strategy for treatment of TNBC and potentially other cancers.

**Electronic supplementary material:**

The online version of this article (10.1186/s12935-019-0740-2) contains supplementary material, which is available to authorized users.

## Background

Approximately 15–20% of all breast cancer deaths in the U.S. occur in patients diagnosed with triple-negative breast cancer (TNBC), a subtype defined by a lack of the estrogen receptor, progesterone receptor, and human epidermal growth factor receptor 2 (Ref. [1]). Due to lack of these targetable cell surface receptors, radiation, surgery, and chemotherapy remain the current standard of care [1, 2]. While a subset of TNBC patients respond initially to chemotherapy, they often suffer from toxicity and acquired resistance, resulting in cancer recurrence and metastasis [2–4]. Therefore, there is a critical need for efficacious therapies that limit toxicity and overcome resistance for TNBC patients.

Recent efforts to improve clinical efficacy of chemotherapies have shifted towards combining them with targeted approaches to lower effective chemotherapeutic doses while maintaining therapeutic response [1, 2]. For instance, in clinical trials that targeted the epidermal growth factor receptor (EGFR), which is commonly upregulated/activated in TNBC, there was no improvement in chemotherapeutic efficacy [5–7]. Moreover, while ongoing clinical trials are testing PI3K–AKT and MEK–ERK signaling pathways downstream of EGFR, toxicity remains unresolved with a modest to no increase in patient survival (NCT02423603, NCT01964924).

Our lab previously found that calcium and integrin-binding protein 1 (CIB1), an intracellular protein that regulates highly oncogenic PI3K–AKT and MEK–ERK signaling, may represent a viable target in TNBC [8, 9]. We showed that CIB1 depletion simultaneously inhibits AKT and ERK activation and induces significant cell death in approximately 70% of TNBC cell lines tested and in an in vivo xenograft model [8]. Common to TNBC cell lines that are sensitive to CIB1 depletion is elevated AKT activation. Thus, CIB1 depletion does not cause cell death in TNBC and normal cell lines that exhibit low basal AKT activity [8]. Here, we evaluated cell death in TNBC versus normal breast epithelial cell lines using novel combination treatments involving CIB1 depletion with and without the commonly used chemotherapeutic docetaxel. We found that CIB1 depletion enhanced docetaxel-induced cell death selectively in TNBC over normal cells largely via a death receptor-mediated, as opposed to mitochondrial-mediated apoptosis.

TNF-related apoptosis-inducing ligand (TRAIL), a death receptor ligand, was once thought to be a promising anti-cancer agent because of its selectivity for killing tumor but not normal cells; however, it was not pursued due to innate or acquired resistance driven by dysfunctional TRAIL receptors [10, 11]. Interestingly, there is a growing interest in combining chemotherapeutic drugs or targeted approaches with TRAIL to overcome this resistance [12–14]. Here we find that CIB1 depletion in combination with the death receptor ligand TRAIL potentiates TNBC-selective cell death, likely due to the upregulation of TRAIL receptor-2 (TRAIL-R2/DR5) in CIB1-depleted TNBC cells. Thus, TRAIL in combination with CIB1 depletion may represent a novel mechanism to sensitize TRAIL-resistant TNBC cells.

Chemotherapy not only causes toxicity, but also often leads to cancer recurrence [3]. Since recurrence driven by drug resistance is often associated with dysfunctional apoptotic mechanisms [15], one approach to circumvent resistance is to induce non-apoptotic cell death [4, 16]. Here, we identified paraptosis as a likely non-apoptotic mode of cell death induced by CIB1 depletion, due to the observed cellular swelling and intracellular vacuolization that are morphological hallmarks of paraptosis [17, 18]. Currently, there are no well-defined molecular mechanisms known to regulate paraptosis. However, the intracellular protein ALG-2-interacting protein X (Alix) has been shown to inhibit paraptosis by preventing cytoplasmic vacuolization, potentially by regulating endosomal sorting and fusion with other organelles [17, 19]. Separate studies showed that known inducers of paraptosis such as withaferin A and reactive oxygen species decrease the expression of Alix in breast cancer cells [18, 20]. Therefore, loss of Alix expression is considered a molecular marker of paraptosis [17, 18, 20, 21]. In addition to downregulation of Alix, insulin-like growth factor I receptor (IGF-1R) tyrosine kinase and JNK activity were found to induce paraptosis [17]. Here we report that CIB1 depletion alone or in combination with docetaxel/TRAIL not only restores apoptotic signaling but also induces paraptosis in docetaxel-resistant TNBC cells. Combination therapies involving CIB1 targeting could therefore provide safe and durable strategies for treatment of TNBC and potentially other cancers.

## Materials and methods

### Cell lines

The human triple-negative breast cancer cell lines (MDA-MB-436 [Perou Lab, UNC], MDA-MB-468 [UNC Lineberger Tissue Culture Facility], and MDA-MB-231 (Otey Lab, UNC) were cultured in Dulbecco’s modified eagle medium (DMEM, Gibco) supplemented with 10% fetal bovine serum (Gemini) and 1% MEM non-essential amino acids (Gibco), with the addition of 10 μg/ml insulin for MDA-MB-436 cells. The human normal breast epithelial cell line (ME16C, Perou lab) was cultured in MEBM (Lonza). All cells were maintained at 37 °C in a humidified atmosphere of 5% CO_2_.

### Generation of docetaxel-resistant MDA-MB-436 TNBC cell line

Docetaxel-resistant MDA-MB-436 cells (MDA-436-DCX^R^) were established by culturing in growth media supplemented with increasing concentrations of docetaxel (5 nM to 50 nM) over a 9-month period. Cells at approximately 40–50% confluency were incubated with a selected concentration for 48 h followed by recovery in drug-free growth media. The concentration of docetaxel was incrementally increased until the cells were resistant, at a final concentration of 50 nM docetaxel (MDA-436-DCX^R^). Parental MDA-MB-436 cells (MDA-436-PR) were cultured and passaged in parallel in drug-free growth media to control for extended time in culture.

### Reagents

Docetaxel (Tocris), recombinant TRAIL (PeproTech), z-VAD-fmk (Enzo Life Sciences), TRAIL-R2 (DR5) neutralizing antibody (R&D system), and human control IgG (Jackson Immuno Research) were used to treat the cell lines.

### CIB1 targeting via RNA interference in the absence and presence of docetaxel or TRAIL

Construction of control and CIB1 shRNA lentiviral plasmids has been described previously [8, 9]. Two different CIB1 targeting sequences (shCIB1-1 and shCIB1-2) were used to validate shRNA-induced CIB1 depletion. To knock down CIB1, cells of interest at ~ 30% confluence were infected with lentiviral shRNA containing 6 µg/ml polybrene (Sigma) for 16–18 h followed by replacement with fresh growth media. After 24 h, the shRNA-infected cells were treated with vehicle, docetaxel, or TRAIL for an additional 48 h. Treated cells were then harvested 4 days post-infection, during which time transduction efficiency exceeds 90% as determined by GFP fluorescence.

### Trypan blue exclusion assay

Cell death was determined by trypan blue exclusion using automated fluorescently activated cell sorting (FACS) counting. Aliquots of 10 μl of both adherent and floating cell populations were collected and stained with 0.004% trypan blue (1:10 dilution). Stained dead cells were selected and counted using the Per-CP (APC) channel [22]. Quantification of total live and dead cell populations was determined using an Accuri C6 Flow Cytometer (BD Biosciences) and data are presented as percent cell death.

### TRAIL-R1 and -R2 fluorescence detection

To detect surface expression of TRAIL-R1 and -R2, 2 × 10^5^ cells were detached using 2 mM EDTA, washed in PBS, and resuspended in PBS (0.1% BSA) containing antibodies for TRAIL-R1/DR4, TRAIL-R2/DR5 (eBiosciences) or IgG control. AlexaFluor 647 (Invitrogen) secondary antibody was used to detect the levels of TRAIL-R1/2, and mean fluorescence intensity was measured via Accuri C6 Flow Cytometer.

### Western blotting

Cells were harvested and lysed with buffer containing 10 mM CHAPS as previously described [8]. Equal amounts of protein based on total cell number were separated by SDS-PAGE, transferred to PVDF membrane, and incubated with primary antibodies overnight at 4 °C. Secondary HRP-conjugated antibodies against rabbit, mouse, and chicken were then used to visualize the immunoblots via enhanced chemiluminescence (ECL2, Pierce). The following antibodies were used in this study: PARP, GAPDH, cleaved caspase-8, caspase-8, cleaved caspase-9, caspase-9, TRAIL-1/DR4, TRAIL-R2/DR5, IGF-1R, phospho-JNK, and total JNK (Cell Signaling), CIB1 (chicken polyclonal antibody, [9]), vinculin (Sigma), Alix (Biolegend).

### Mitochondrial membrane potential detection

To detect changes in mitochondrial membrane potential, cells were stained with JC-1 (Thermofisher), a cationic dye that accumulates in the mitochondria. Cells (1 × 10^6^) were dissociated and incubated in PBS containing JC-1 (5 µM) for 30 min at 37 °C. JC-1 accumulates in functional mitochondria to form aggregates that fluoresce red. In dysfunctional mitochondria with decreased membrane potential, JC-1 will instead remain as monomers that fluoresce green. After washing with PBS, the emission of JC-1 fluorescence was analyzed using an Accuri C6 Flow Cytometer and associated software (BD Biosciences). Therefore, calculation of red:green JC-1 fluorescence ratio was used as the surrogate for changes in mitochondrial membrane potential.

### Microscopy imaging

To assess morphological changes induced by CIB1 depletion in the absence and presence of docetaxel or TRAIL, cells were imaged in 6-well plates at the conclusion of each experiment. Differential interference contrast (DIC) images were captured using a Nikon TE300 microscope equipped with an Andor Zyla sCMOS camera (20x objective).

### Statistics

The statistically significant differences were determined using either a one-way ANOVA or student’s two-tailed t-test. More details on p-values are indicated in the figure legends.

## Results

### CIB1 depletion selectively enhances docetaxel-induced TNBC cell death

We previously reported that CIB1 depletion leads to TNBC cell death while sparing normal cells [8], suggesting that CIB1 may be a viable, safe target. To address the need to enhance tumor cell death and maintain tolerability by normal cells, we tested the targeting of CIB1 via RNA interference in combination with the commonly used chemotherapeutic agent, docetaxel, in both TNBC and a normal breast epithelial cell line. Two different CIB1 shRNA sequences (shCIB1-1 and shCIB1-2) were used to validate CIB1 depletion (Additional file 2: Figure S1A). CIB1 depletion-induced cell death quantification, cell death signaling, and cellular morphology were also validated using both shRNA sequences (Additional file 2: Figure S1B–H) and Western blots were quantified in Additional file 1: Table S1. Since similar results were observed with both shRNA sequences, we chose CIB1 shRNA-1 for the remainder of the experiments.

**Fig. 1:**
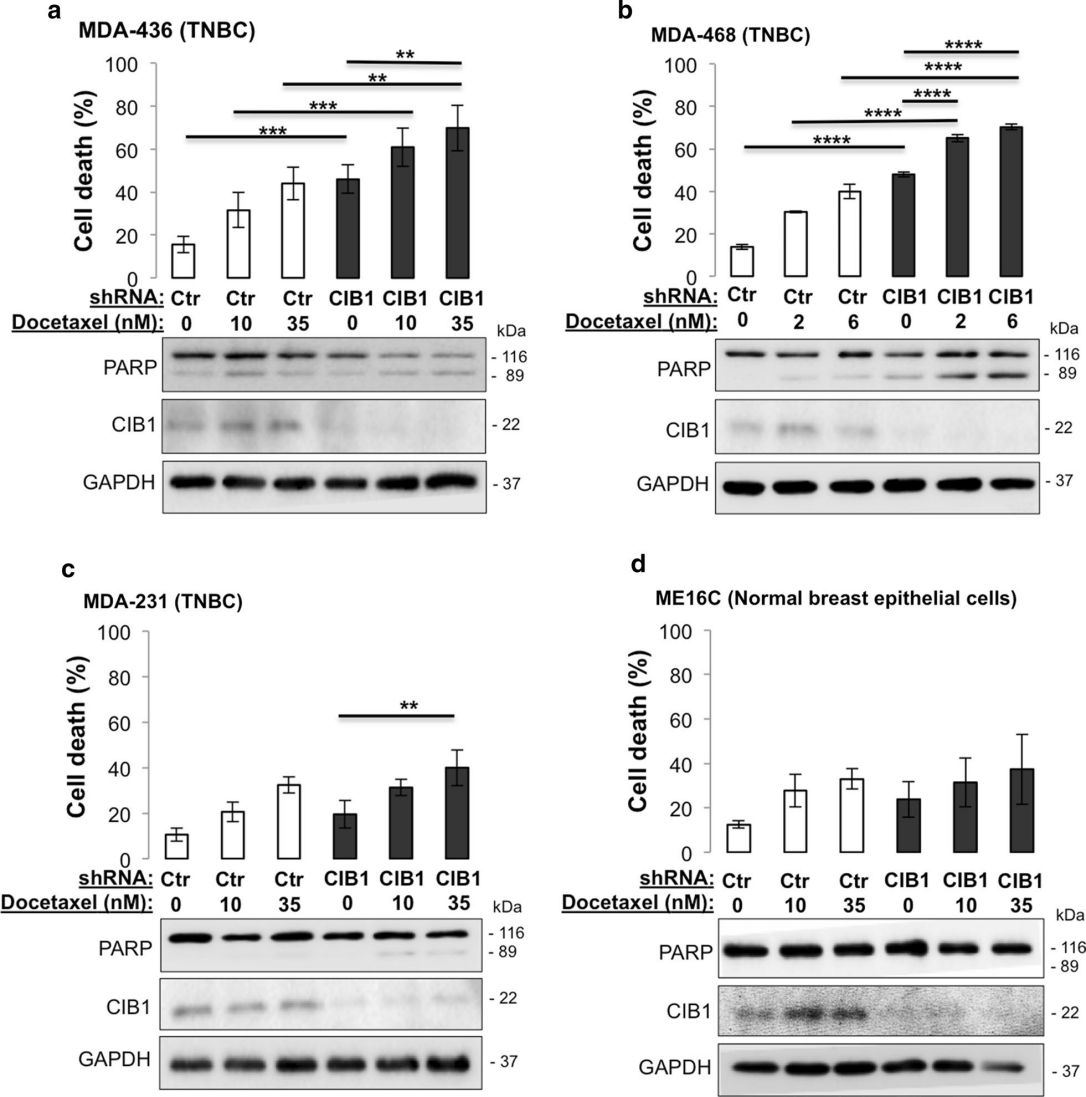
CIB1 depletion in combination with docetaxel enhances cell death and increases caspase activation in TNBC but not normal ME16C cells. Combination of CIB1 depletion with docetaxel in both TNBC and normal cells was tested. Cells were infected with control or CIB1 shRNA for 2 days before addition of vehicle (DMSO) or the indicated concentrations of docetaxel for 48 h. Percent cell death in **a** MDA-436 TNBC (n = 5), **b** MDA-468 TNBC (n = 3), **c** MDA-231 TNBC (n = 3), and **d** ME16C normal breast epithelial cells (n = 4) was quantified via a trypan blue exclusion assay from both adherent and floating cell populations. Lysates prepared from cells in **a**–**d** [n = 5; 3; 3; 4, respectively] were analyzed by Western blotting with the indicated antibodies (lower panels). Data represent mean ± SD (*P < 0.05, **P < 0.01, ***P < 0.001, and ****P < 0.0001, ANOVA)

To test whether the combination of CIB1 depletion and docetaxel enhances cell death, we selected three different TNBC cell lines based on their sensitivity to CIB1 depletion-MDA-MB-436 (sensitive, Fig. 1a), MDA-MB-468 (sensitive, Fig. 1b), and MDA-MB-231 (insensitive, Fig. 1c) [8]. Dosages of docetaxel that caused 30–50% TNBC cell death were chosen to be combined with CIB1 depletion (Fig. 1). We found that CIB1 depletion enhanced docetaxel-induced cell death more effectively in CIB1 depletion-sensitive (Fig. 1a, b) versus insensitive cells (Fig. 1c). The enhanced TNBC MDA-MB-436 cell death was indistinguishable with either shCIB1-1 or -2 alone or combined with docetaxel (Additional file 2: Figure S1B). In contrast, CIB1 depletion alone or in combination with docetaxel did *not* significantly increase cell death in normal breast epithelial ME16C cells (Fig. 1d), suggesting that CIB1 targeting may improve chemotherapeutic efficacy by tumor-selective killing.

To elucidate the mechanism underlying the enhanced TNBC cell death induced by CIB1 depletion plus docetaxel (Fig. 1a–c), we analyzed cell lysates for cleaved PARP, a marker of apoptosis. In agreement with the observed enhanced cell death, the combined treatment-induced PARP cleavage was only observed in TNBC (Fig. 1a–c) but not normal breast epithelial ME16C cells (Fig. 1d). CIB1 knockdown was confirmed by Western blotting (Fig. 1a–d). Collectively, our results indicate that the novel combination of CIB1 depletion and docetaxel selectively enhances TNBC cell death via increased apoptotic signaling.

### CIB1 depletion with docetaxel induces death receptor-mediated apoptosis

**Fig. 2:**
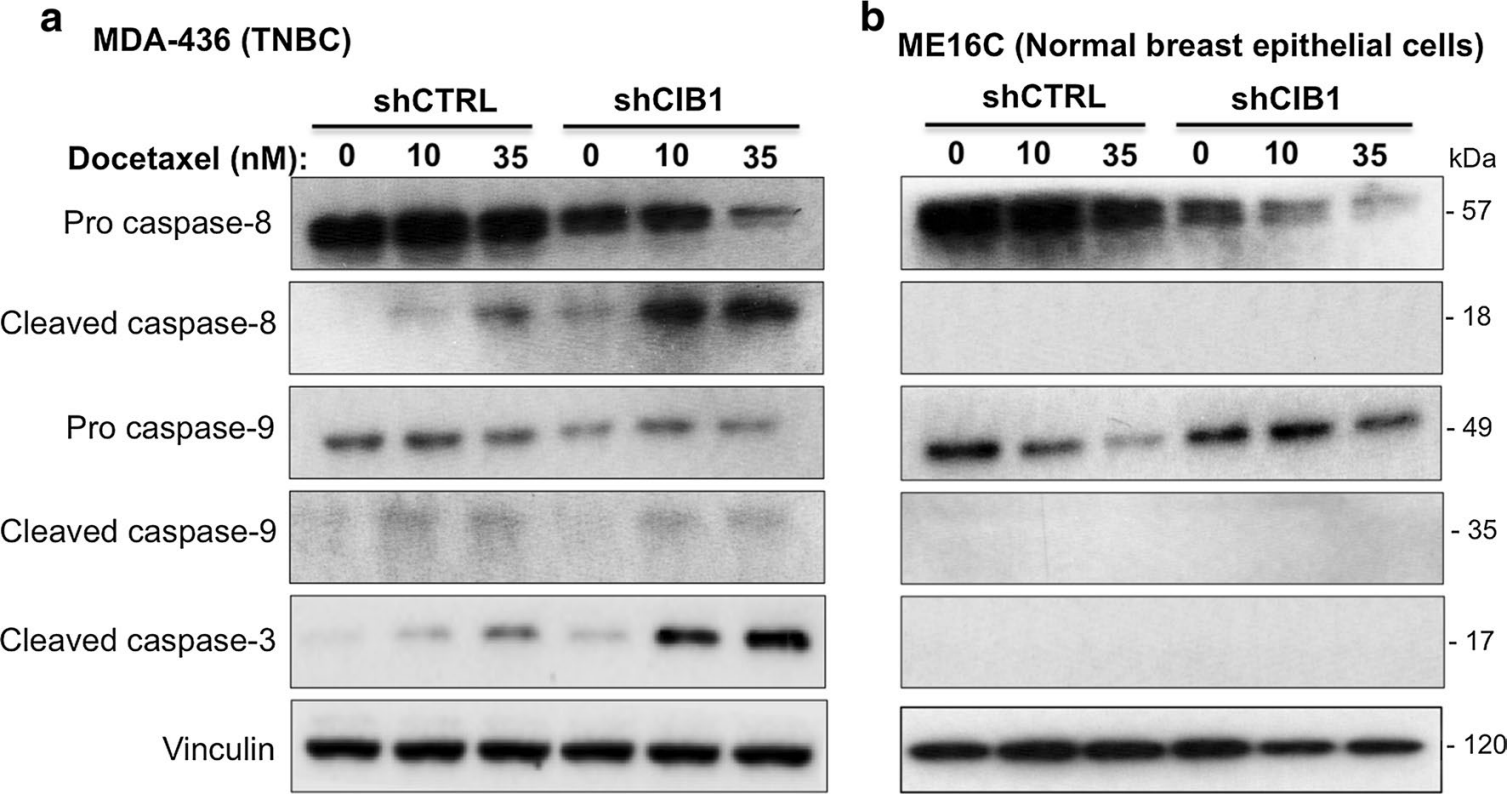
CIB1 depletion combined with docetaxel enhances death receptor-mediated apoptotic signaling. Docetaxel-induced caspase-8 and -3 activation is enhanced in CIB1-depleted MDA-436 but not normal ME16C cells. Control or CIB1-depleted **a** MDA-436 TNBC (n = 5) and **b** ME16C normal breast epithelial cells (n = 4) were treated with docetaxel as in Fig. 1. Lysates prepared from each cell line were probed for pro-caspase-8, cleaved caspase-8, pro-caspase-9, cleaved caspase-9, cleaved caspase-3, CIB1, and vinculin (loading control)

To further understand the apoptotic signaling mechanisms induced by CIB1 depletion in combination with docetaxel, we asked whether death receptor-mediated or mitochondrial apoptosis, which are initiated by caspase-8 and caspase-9, respectively, contributed to the observed cell death. We detected degradation of inactive pro-caspase-8 and the appearance of active or cleaved caspase-8, but not caspase-9, in CIB1-depleted cells treated with docetaxel (Fig. 2a and Additional file 2: Figure S1C). In contrast, we detected minimal caspase-8 activity in CIB1-depleted cells alone (Fig. 2a and Additional file 2: Figure S1C), suggesting that CIB1 depletion may prime TNBC cells for death receptor-mediated apoptosis. Comparable effects on apoptotic signaling induced by CIB1 depletion alone or with docetaxel was also observed using CIB1 shRNA-2 (Additional file 2: Figure S1C). We additionally probed for caspase-10, a controversial marker of death receptor-mediated apoptosis [23], and found no further caspase-10 activation upon combination treatments (data not shown). Caspase-3, a downstream substrate of caspase-8, was also activated by the combination treatment (Fig. 2a). In contrast, we did *not* observe caspase activation in control or CIB1-depleted ME16C normal breast epithelial cells with or without docetaxel (Fig. 2b). CIB1 depletion in combination with docetaxel also selectively activated caspase-8 in two additional TNBC cell lines, MDA-468 (Additional file 3: Figure S2A) and MDA-231 cells (Additional file 3: Figure S2B). Collectively, these results suggest that death receptor-mediated apoptotic signaling contributes mechanistically to enhanced cell death induced by the combination of CIB1 depletion and docetaxel selectively in TNBC cells, regardless of their sensitivity to CIB1 depletion.

### CIB1 depletion sensitizes TNBC, but not normal cells to TRAIL

**Fig. 3:**
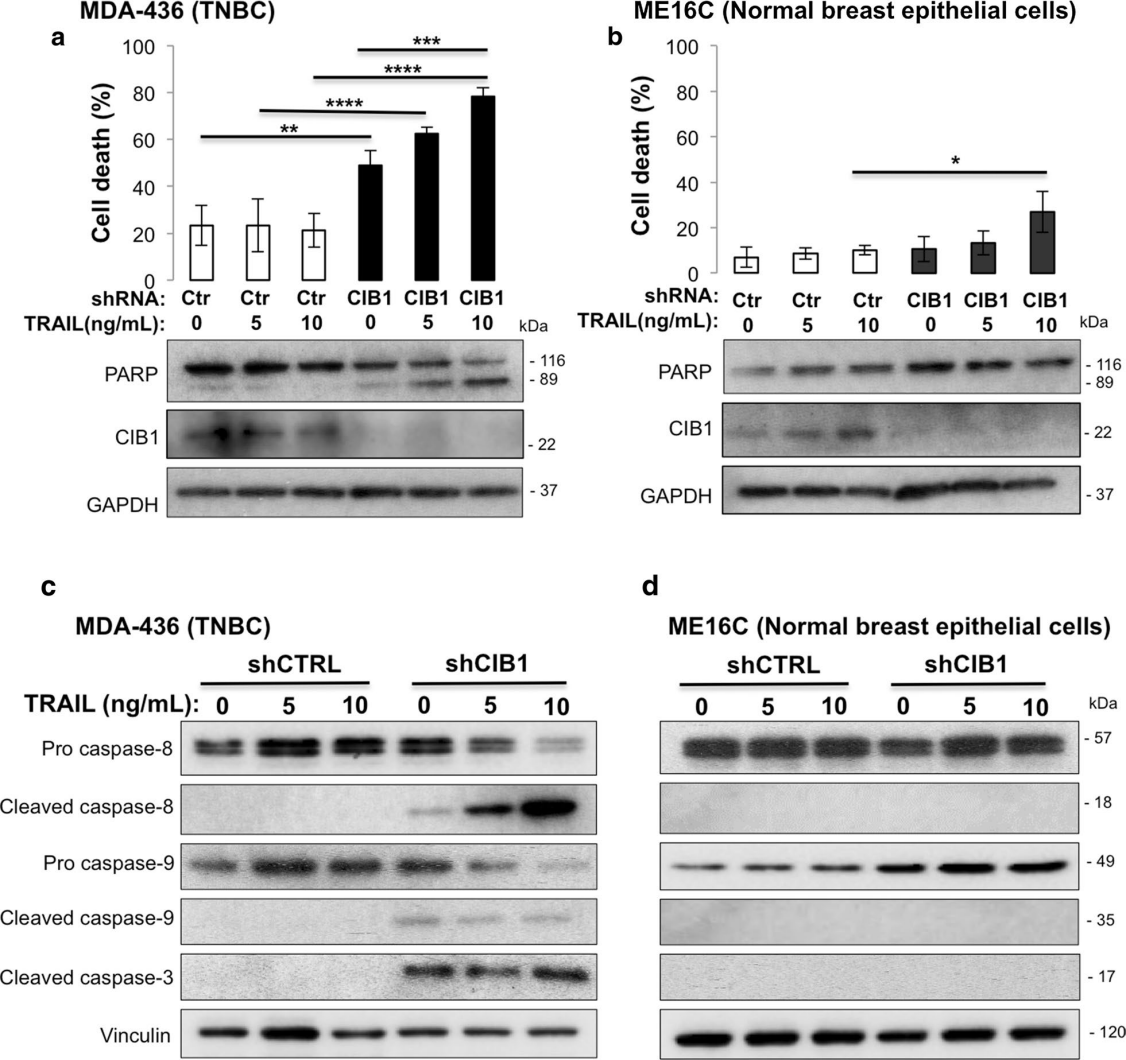
CIB1 depletion sensitizes MDA-436 TNBC but not normal ME16C cells to TRAIL. Combination of CIB1 depletion with TRAIL in both TNBC and normal cells was tested. Cells were infected with control or CIB1 shRNA for 2 days before the addition of vehicle (water) or the indicated concentrations of a death receptor ligand TRAIL for 48 h. Percent cell death quantified via trypan blue exclusion using **a** MDA-436 TNBC (n = 4) and **b** ME16C normal breast epithelial cell lines (n = 3). Data represent mean ± SD (*P < 0.05, **P < 0.01, ***P < 0.001, and ****P < 0.0001, ANOVA). CIB1 depletion plus TRAIL enhanced caspase-8 and -3 activation. Representative Western blot for pro-caspase-8, cleaved caspase-8, pro-caspase-9, cleaved caspase-9, cleaved caspase-3, CIB1, and vinculin in **c** MDA-436 TNBC (n = 3) and **d** ME16C cells (n = 3)

Since CIB1 depletion enhances docetaxel-induced TNBC cell death via increased death receptor signaling, we investigated the link between CIB1 depletion and two cytotoxic death receptors, TRAIL (TRAIL-R) and Fas. Treatment of CIB1-depleted MDA-436 cells with Fas ligand had no effect on cell death or caspase-8 activation (data not shown), and therefore was not pursued further. However, treatment with TRAIL caused a dose-dependent increase in PARP cleavage (Fig. 3a). Notably, CIB1 depletion sensitized MDA-436 cells to TRAIL, resulting in a synergistic increase in cell death (Fig. 3a and Additional file 2: Figure S1D). In contrast, CIB1 depletion plus TRAIL failed to induce PARP cleavage and cell death in normal ME16C cells (Fig. 3b), indicating that this combination is highly selective in killing TNBC while sparing normal cells. Further, the enhanced cell death correlated with active or cleaved caspase-3 and caspase-8, but not caspase-9, in CIB1 shRNA-1 and shRNA-2 depleted MDA-436 TNBC cells (Fig. 3c and Additional file 2: Figure S1E) not ME16C cells (Fig. 3d). Caspase-8 activation was also confirmed by the corresponding degradation of inactive pro-caspase-8 (Fig. 3c). Although we did not detect caspase-9 activation (Fig. 3c), staining with the mitochondrial dye JC-1 revealed increased mitochondrial dysfunction in CIB1-depleted cells alone and in combination with both TRAIL and docetaxel (Additional file 4: Figure S3A). Caspase-8 can cleave the pro-apoptotic Bcl-2 member, Bid, which is an important mediator of mitochondrial dysfunction [24]. Consistent with our JC-1 results, we detected increased Bid cleavage in CIB1 depleted cells treated with either docetaxel or TRAIL (Additional file 4: Figure S3B, C), further suggesting apoptotic cell death.

Interestingly, we also observed a similar increase in caspase-8 activation and cell death upon addition of TRAIL to CIB1-depleted MDA-468 (Additional file 5: Figure S4A) but not MDA-231 TNBC cells (Additional file 5: Figure S4B). This suggests that the cell death induced by the combination of CIB1 depletion and TRAIL, unlike that induced by docetaxel, is cell-type specific based on sensitivity to CIB1 depletion. Thus, adding TRAIL to CIB1 depletion-sensitive cells is a potent combination for inducing caspase-8 activation and cell death.

### TRAIL-R2 upregulation by CIB1 depletion sensitizes TNBC cells to TRAIL

**Fig. 4:**
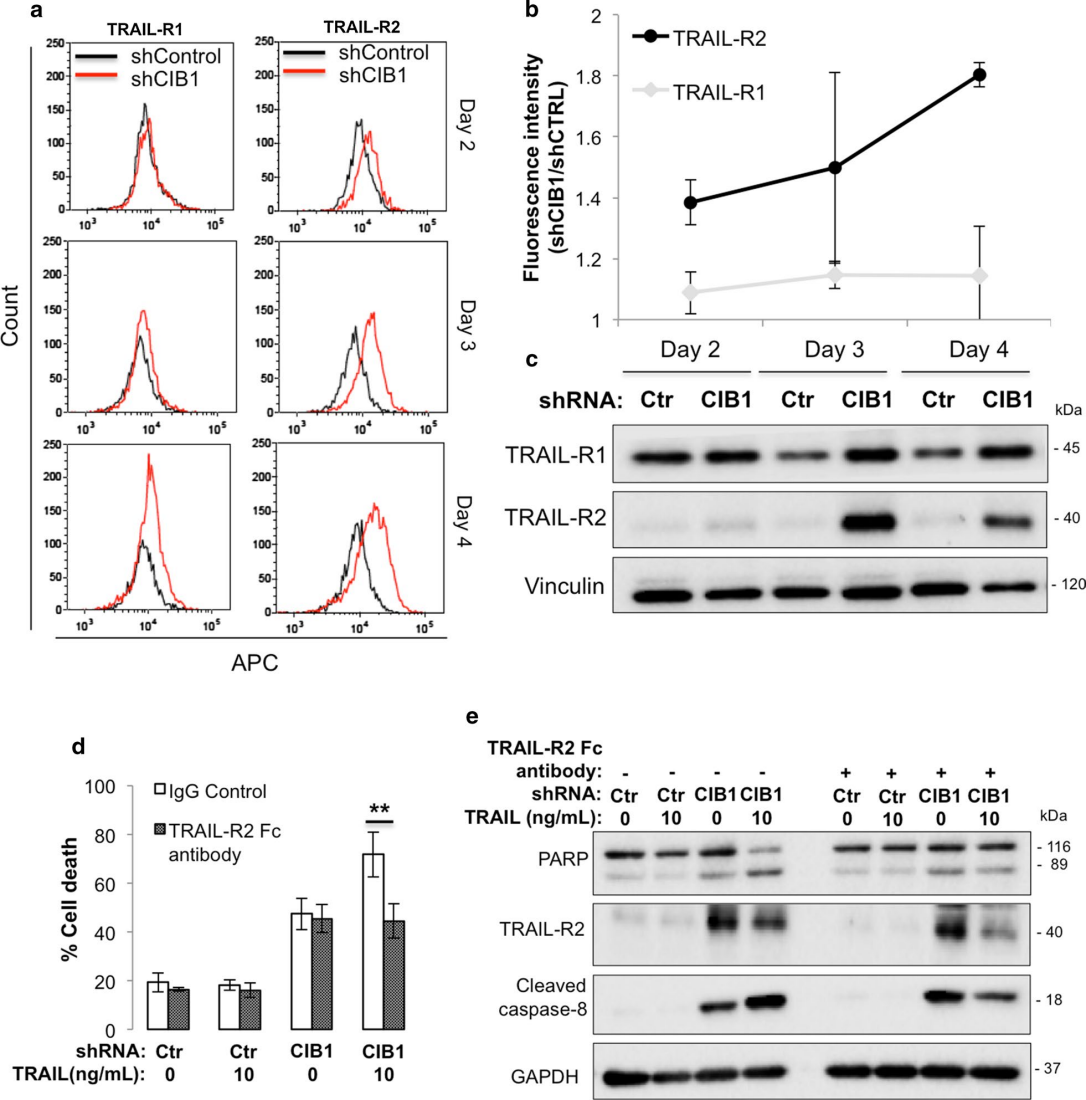
CIB1 depletion upregulates TRAIL-R2 expression Death receptors TRAIL-R1/R2 levels in MDA-436 cells were measured at Days 2, 3, and 4 post-infection with either control or CIB1 shRNA. **a** FACS analysis shows upregulation of cell surface levels of TRAIL-R2, but not TRAIL-R1, starting at 2, 3 and 4 days post-infection (2nd and 3rd row) with CIB1 shRNA (red) relative to control (black). **b** Mean ± SD (n = 3) of fluorescence intensities of CIB1 shRNA-infected MDA-436 cells normalized to control cells shown in **a**. **c** Representative western blot (n = 3) showing increased whole cell expression of TRAIL-R2, but not TRAIL-R1, in CIB1-depleted MDA-436 cells at 3 and 4 days post-infection with CIB1 shRNA. **d** TRAIL-R2 neutralizing antibody blocks TRAIL-induced cell death in CIB1-depleted cells. Control or CIB1-depleted MDA-436 cells were pre-treated with either IgG control or TRAIL-R2 neutralizing antibody (TRAIL-R2 Fc, 1 ng/ml) for 24 h before addition of vehicle (water) or 10 ng/ml recombinant TRAIL ligand for 48 h (n = 3). Cell death was quantified as in Fig. 1 (**P < 0.01). **e** Blocking TRAIL-R2 inhibits TRAIL-induced PARP cleavage and caspase-8 activation in CIB1-depleted MDA-436 cells. Representative Western blot of PARP, TRAIL-R2, cleaved caspase-8, CIB1, and GAPDH (loading control) in treatment groups shown in d) (n = 3)

Because CIB1 depletion sensitizes TNBC cells to TRAIL, we asked whether this effect was due to increased TRAIL-R expression. We measured cell-surface and whole cell expression of TRAIL-R1 (DR4) and/or -R2 (DR5) using flow cytometry and Western blotting, respectively. We found that TRAIL-R2, but not TRAIL-R1, was upregulated on the surface of CIB1-depleted versus control MDA-436 TNBC cells, starting 2 days post-infection with both CIB1-1 and CIB1-2 shRNA sequences (Fig. 4a, b; Additional file 2: Figure S1F, G). This was notable because TRAIL was added 2 days post-infection and coincides with acquired sensitivity to TRAIL. Whole cell expression levels of TRAIL-R2 also increased with CIB1 depletion starting at Day 2, but more significantly at Day 3 post-infection (Fig. 4c). Next, to confirm that TRAIL-R2 upregulation upon CIB1 depletion was responsible for sensitizing MDA-436 cells to TRAIL, we blocked TRAIL-R2 function by pre-treating control or CIB1-depleted MDA-436 TNBC cells with a neutralizing (TRAIL-R2 Fc) antibody. Blocking TRAIL-R2 prevented the increased cell death induced by TRAIL in CIB1-depleted cells (Fig. 4d). Further, this rescue of cell viability by the TRAIL-R2 neutralizing antibody was also associated with decreased PARP cleavage and caspase-8-activation, whereas the upregulation of TRAIL-R2 by CIB1 depletion remained intact (Fig. 4e). These results indicate that CIB1 depletion-induced TRAIL-R2 upregulation sensitizes TNBC cells to TRAIL.

### CIB1 depletion plus docetaxel or TRAIL induces caspase-independent cell death

**Fig. 5:**
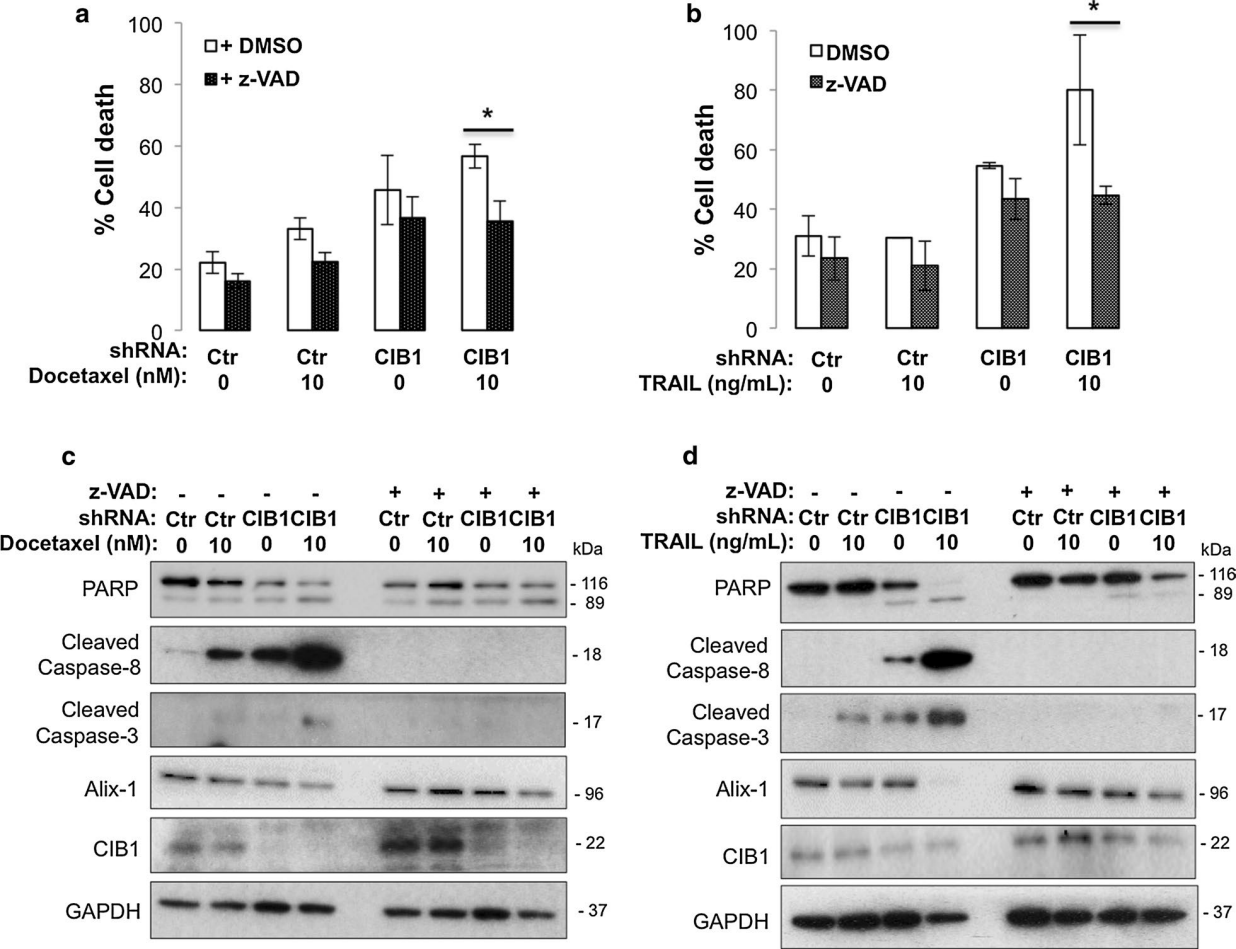
CIB1 depletion in combination with docetaxel or TRAIL induces caspase-independent cell death. Pre-treatment using a pan-caspase inhibitor, z-VAD-fmk (z-VAD) helped to determine contributions of caspase-independent, non-apoptotic cell death mechanisms induced by the combination treatments. Control or CIB1-depleted cells were pretreated with vehicle (DMSO) or 50 µM z-VAD-fmk (z-VAD) for 24 h before adding **a** 10 nM docetaxel or **b** 10 ng/ml TRAIL for 48 h. The concentrations for docetaxel and TRAIL were chosen because we observed a sufficient increase in cell death and caspase-8 activity when combined with CIB1 depletion. Percent cell death was quantified as in Fig. 1, represented by mean ± SD, *P < 0.05 (n = 3). z-VAD inhibits caspase-8 but does not affect PARP activation or the expression of a marker of paraptosis, Alix. Lysates prepared from control or CIB1-depleted cells in **a** and **b** treated with **c** docetaxel or **d** TRAIL were probed with for PARP, cleaved caspase-8, cleaved caspase-3, Alix, CIB1, and vinculin (loading control). Western blot is representative of 3 independent experiments

To confirm apoptosis as the primary mechanism underlying the observed death of CIB1-depleted MDA-436 TNBC cells with or without docetaxel/TRAIL, we pre-treated cells with the pan-caspase inhibitor, z-VAD-fmk (z-VAD). Surprisingly, we found that z-VAD partially rescued MDA-436 cell viability induced by CIB1 depletion in combination with either docetaxel (Fig. 5a) or TRAIL (Fig. 5b) despite complete inhibition of caspase-8 and caspase-3 activation (Fig. 5c, d). These results led us to investigate the contribution of caspase-*independent* cell death mechanisms. Phenotypically, we noticed that CIB1 depletion alone induced cellular swelling and intracellular vacuolization (Additional file 2: Figure S1H), which are characteristic of autophagic, necroptotic, or paraptotic modes of caspase-independent non-apoptotic cell death [16, 17, 25]. After detecting no changes in LC-III and p-RIPK1 levels, markers of autophagy and necroptosis, respectively (data not shown), those modes of cell death seemed less likely. We then assessed ALG-2-interacting protein X (Alix) protein expression levels, which correlate inversely with induction of paraptosis [17, 18]. We detected decreased Alix expression upon CIB1 depletion in combination with either docetaxel (Fig. 5c) or TRAIL (Fig. 5d) (0.3- or 0.08-fold respectively). Even with z-VAD pre-treatment, we detected decreased Alix expression in response to CIB1 depletion plus docetaxel or TRAIL (0.6- or 0.7-fold respectively, Fig. 5c, d), suggesting paraptosis as a non-apoptotic component of cell death. Because paraptosis can also be mediated by IGF-1R signaling and JNK activation [17] we probed for IGF-1R expression and phospho-JNK. We found that CIB1 depletion alone led to increased IGF-1R expression, and the combination treatment activated JNK signaling, further supporting the involvement of paraptosis (Additional file 6: Figure S5A). Although not a specific inhibitor of paraptosis (protein synthesis inhibitor), pre-treatment using cycloheximide rescued CIB1 depleted TNBC cells (Additional file 6: Figure S5B). These findings suggest that CIB1 depletion combined with docetaxel or TRAIL induces paraptosis.

### Docetaxel-resistant TNBC cells remain sensitive to CIB1 depletion alone or in combination with docetaxel or TRAIL

**Fig. 6:**
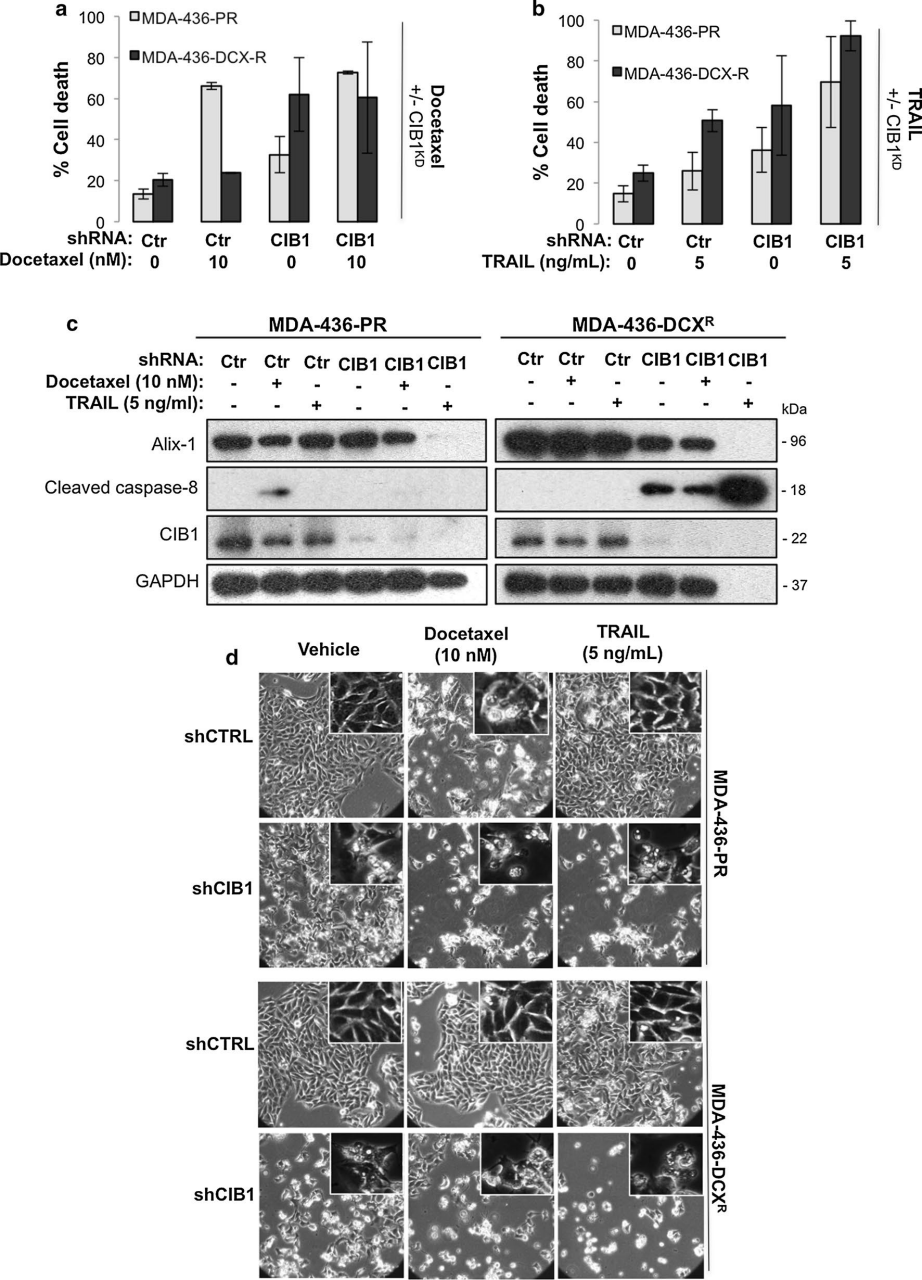
Docetaxel resistant MDA-436 cells are sensitive to CIB1 depletion alone and in combination with docetaxel or TRAIL. To determine whether chemo-resistance is overcome by the combination treatments, cell death and its mechanism were analyzed. Control or CIB1-depleted MDA-436-PR (parental) and MDA-436-DCX^R^ (docetaxel-resistant) cells were treated with either **a** vehicle control (DMSO) or 10 nM docetaxel (n = 3), **b** or 5 ng/ml TRAIL ligand (n = 3) for 48 h. Percent cell death shown in mean ± SD was quantified as in Fig. 1. **c** Western blots showing increased caspase-8 activation and decreased Alix expression in CIB1-depleted MDA-436-DCX^R^ cells alone or in combination with docetaxel or TRAIL, representative of 3 individual experiments. GAPDH was used as a loading control. **d** Representative DIC images (20×) of control or CIB1-depleted parental (MDA-436-PR) and MDA-436-DCX^R^ cells treated with either vehicle or 10 nM docetaxel or 5 ng/ml. Insets show characteristics of paraptotic morphology in CIB1-depleted cells in the absence or presence of docetaxel/TRAIL

Defective apoptosis is one mechanism of resistance and a major obstacle for effective chemotherapy [15]. Therefore, we asked whether CIB1 depletion overcomes resistance by restoring apoptosis. To answer this question, we generated docetaxel-resistant MDA-MB-436 cells (MDA-436-DCX^R^). Resistance was confirmed by comparing the EC_50_ of docetaxel in parental (MDA-436-PR; 3 nM) versus resistant (MDA-436-DCX^R^; 219 nM) cells (Additional file 7: Figure S6A). CIB1 depletion alone or with docetaxel (Fig. 6a) or TRAIL (Fig. 6b) induced significant cell death in docetaxel-resistant MDA-436-DCX^R^ cells. Western blotting analysis showed that although docetaxel-induced caspase-8 activity is compromised in MDA-436-DCX^R^ resistant cells, CIB1 depletion alone and in combination with docetaxel restored caspase-8 activity in these cells (Fig. 6c). Contrary to previous findings, caspase-8 activity was not detected in CIB1-depleted MDA-436-PR parental cells with or without docetaxel/TRAIL (Fig. 6c). This may be due to alterations in caspase activities [26] resulting from extended culturing of MDA-436–PR cells during the process of establishing MDA-436-DCX^R^ cells (> 9 months with > 60 passages). Unexpectedly, CIB1 depletion plus TRAIL caused nearly complete death of MDA-436-DCX^R^ cells (Fig. 6b), which may explain an apparent lack of GAPDH expression (Fig. 6c) due to the lysates being composed of mostly floating dead cells. The nearly complete MDA-436-DCX^R^ cell death correlated with a more profound caspase-8 activation (Fig. 6c). This further amplification of caspase-8 activity could be due to increased cell surface expression of both TRAIL-R1 and -R2 on CIB1-depleted MDA-436-DCX^R^ cells as opposed to only TRAIL-R2 in CIB1-depleted MDA-436-PR cells (Additional file 7: Figure S6B, C). Whole cell expression levels of TRAIL-R2 increased with CIB1 depletion in MDA-436-DCX^R^ cells (Additional file 7: Figure S6D). These results suggest that CIB1 depletion alone and in combination with either docetaxel or TRAIL has the potential to overcome chemo-resistance.

Since another strategy to circumvent chemo-resistance is to induce non-apoptotic, caspase-independent cell death [16], we asked whether paraptosis also occurs in docetaxel-resistant cells. Indeed, we found that either CIB1 depletion alone or in combination with docetaxel or TRAIL decreased Alix levels in MDA-436-DCX^R^ cells (Fig. 6c). Moreover, paraptotic morphologies such as swelling and intracellular vacuole formation were observed in resistant cells in response to CIB1 depletion alone or in combination with docetaxel/TRAIL (Fig. 6d). The morphology of CIB1-depleted cells mimicked the effects of docetaxel (Fig. 6d), a parental compound to paclitaxel, which is known to cause paraptotic morphology at high doses [27]. Although the combination treatments did not activate the JNK pathway, CIB1 depletion alone led to upregulation of IGF-1R, an upstream effector of paraptosis, in docetaxel-resistant TNBC cells similar to that observed in parental cells (Additional file 7: Figure S6E). These results collectively show that the combination treatments both restore apoptosis and induce paraptosis as potential mechanisms to overcome chemo-resistance.

## Discussion

The standard of care for TNBC patients, chemotherapy and/or surgery, often fails due to development of resistance and toxicity [3, 28]. To address this need, clinical trials have centered on combining targeted therapies with chemotherapeutics, yet resistance and toxicity persist to limit the overall efficacy [2, 4–6]. Our previous work indicated that CIB1 may be a potentially safe target due to its selectivity for killing TNBC but not normal cells when depleted [8]. Therefore, we tested a novel combination treatment with CIB1 depletion and a commonly used chemotherapeutic, docetaxel to enhance TNBC-selective cell death.

In this study, we focused on detecting and understanding the mechanisms of cell death to assess the combination of CIB1 depletion and chemotherapeutics. Many previous pre-clinical cell culture-based TNBC studies that combine targeted agents with chemotherapeutics have focused primarily on slowing cell proliferation as opposed to cell death [29–32]. Moreover, studies that have quantified cell death were often limited to studying classical caspase-dependent apoptosis [33–36]. In contrast, we investigated both apoptotic and non-apoptotic TNBC cell death in response to CIB1 depletion alone or in combination with docetaxel. In addition, we demonstrate that induction of non-apoptotic cell death is an effective strategy to circumvent resistance, which is often associated with dysfunctional apoptotic signaling [16, 37, 38]. Thus, we propose that quantification of cell death in tumor versus normal cells may identify combinations that eradicate tumors while sparing normal cells, which may be a better indicator for a safer, more efficacious therapy.

Initially, we found that combining CIB1 depletion with docetaxel significantly enhances TNBC cell death while sparing normal breast epithelial cells. The enhanced cell death correlated with increased death receptor-mediated (caspase-8) apoptotic signaling, selectively in TNBC relative to normal cells. Subsequently, we found that the combination of CIB1 depletion and the death receptor ligand TRAIL is potent in selectively killing TNBC cells via increased caspase-8 activation. While TNBC cells are inherently resistant to TRAIL alone, CIB1 depletion appears to sensitize these cells to TRAIL by upregulating TRAIL receptor-2. Once believed to be a safe, tumor-specific targeting therapy, TRAIL as a mono-therapy failed due to resistance driven by mutations in TRAIL receptors and dysfunctional signaling complexes at the receptor intracellular domain [10, 11, 39]. Subsequent studies have focused on combination approaches using chemotherapeutics, natural compounds and targeted agents to sensitize otherwise resistant breast cancer cells to TRAIL by upregulating TRAIL receptors and restoring TRAIL receptors’ intracellular signaling complex activity [14, 40–45]. In comparison, our approach of targeting CIB1 allowed us to use five to tenfold lower concentrations of TRAIL to induce as much or greater cytotoxicity in cultured TNBC cells. The selectivity of TRAIL-based combination treatments for tumor cells is consistent between our results and the published results of other studies [14, 43, 45]. However, those studies provide limited understanding as to why normal cells are unaffected by TRAIL-based combination treatments. We speculate that CIB1 depletion may upregulate decoy receptors, which inhibit intracellular TRAIL receptor signaling and initiate pro-survival NF-κB signaling [46, 47] (data not shown), thereby protecting normal breast epithelial cells from TRAIL-induced cell death. Another mechanism may involve the internalization of TRAIL receptors and expression of intracellular signaling complex modulators such as c-FLIP, XIAP, and IAP in normal cells, to explain TNBC-selective cell death. We believe that a better understanding of cell death mechanisms underlying tumor selectivity versus normal cells could provide further rationale to test a TRAIL-based combination treatment with CIB1 targeting.

The induction of non-apoptotic death could prove to be an effective therapeutic strategy to circumvent resistance [16, 38, 48, 49]. In addition to previously identifying GAPDH nuclear translocation [9], we found paraptosis as another mode of non-apoptotic cell death associated with CIB1 depletion. To our knowledge, we are the first to report paraptosis as a mechanism for overcoming chemoresistance in TNBC cells. While autophagy is another mode of non-apoptotic cell death that shares phenotypic characteristics similar to paraptosis, we did not observe activation of LC-III, a marker of autophagy. Necroptosis, like apoptosis, can be activated downstream of death receptors to cause a caspase-independent paraptotic-like, necrotic phenotype [50]. However, we found no effect on the necrotic marker p-RIPK or the necroptosis inhibitor necrostatin-1 and therefore concluded that CIB1 depletion alone or in combination with either docetaxel or TRAIL does not induce necroptosis. Because pre-treatment with cycloheximide, a protein synthesis inhibitor, rescued CIB1 depleted TNBC cells from cell death, protein synthesis may be investigated to further understand non-apoptotic cell death in relation to CIB1. Future studies examining non-apoptotic modes of cell death associated with CIB1 targeting will provide additional insight into mechanisms to circumvent chemo-resistance.

## Conclusions

**Fig. 7:**
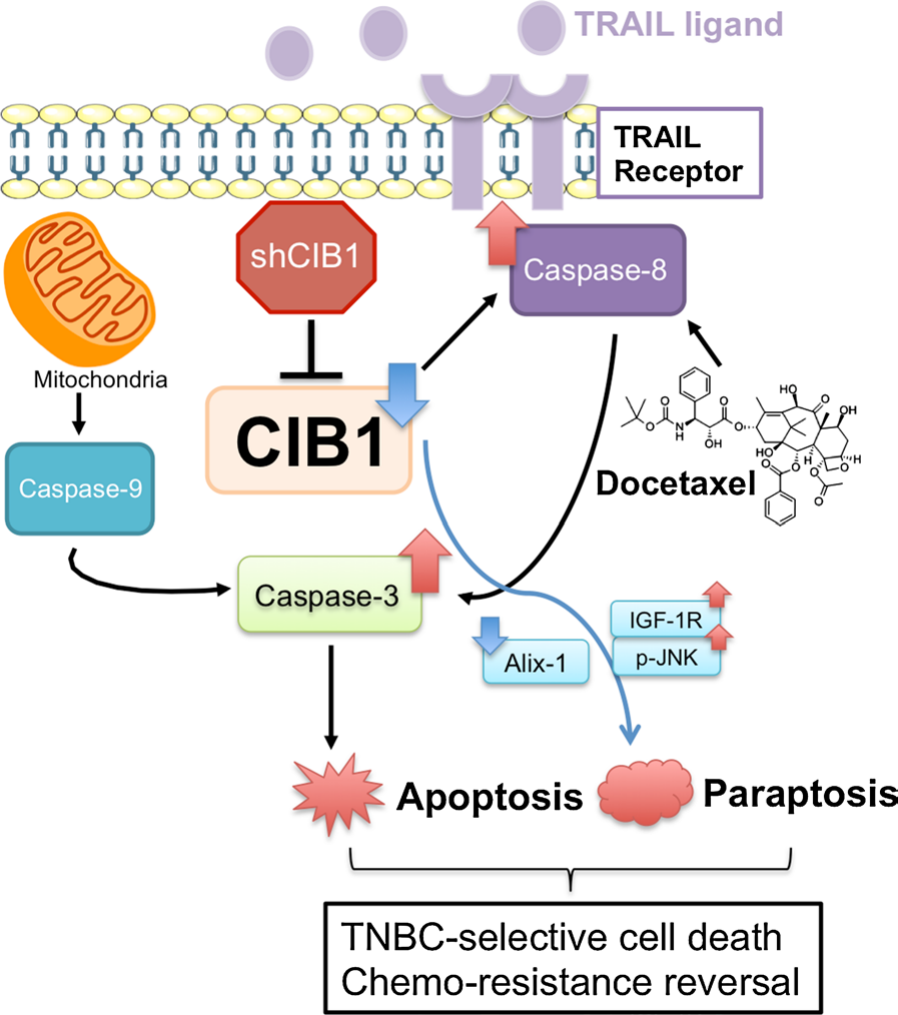
Model of cell death mechanisms induced by CIB1 depletion alone or in combination with docetaxel/TRAIL. The combination of CIB1 targeting via RNA interference and docetaxel/TRAIL activates both apoptotic and non-apoptotic (paraptotic) signaling to induce TNBC-selective cell death and overcome chemo-resistance. Caspase-3/-8 activation and decreased Alix levels were used as markers of apoptosis and paraptosis, respectively

In summary, we find that targeting CIB1 in combination with docetaxel or TRAIL is selective in killing TNBC over normal breast epithelial cells. Also, both combinations restore apoptosis and induce non-apoptotic cell death to overcome resistance (Fig. 7), suggesting well-tolerated and durable treatment options. Hence, we speculate that CIB1 as a target may provide new avenues for formulating effective combination therapies not only for TNBC but other cancers as well.

## Additional files

**Additional file 1: Table S1.** Comprehensive quantification of cell death signaling levels by densitometry.

**Additional file 2: Figure S1.** Validation of CIB1 depletion alone or in combination with docetaxel or TRAIL. MDA-436 TNBC cells were infected with either control or two different lentiviral CIB1 shRNA sequences (FG12 [1] and PLKO [2]). **a)** Western blot results followed by quantification via densitometry show similar CIB1 knockdown and TRAIL-R2 upregulation in MDA-436 cells infected with either CIB1 shRNA-1 or -2 for 96 h. Two days-post infection, cells were treated with vehicle control or, **b)** docetaxel (1 [n=5] and 2 [n=3]), or **c)** TRAIL (1 [n=3] and 2 [n=3]) for 48 h. Percent cell death was quantified via trypan blue exclusion assay and is shown as means +/- SD. Next, we examined death receptor-mediated apoptotic and paraptotic signaling induced by the combination treatment using CIB1 shRNA-1 or -2. Representative Western blot showing PARP, cleaved caspase-9, cleaved caspase-8, Alix, CIB1, and GAPDH in shControl (shCTRL) or shCIB1 (1 and 2) infected cells in combination with **d)** docetaxel (1 [n=5] and 2 [n=3]) or **e)** TRAIL (1 [n=3] and 2 [n=3]). FACS analysis of **f)** TRAIL-R1 and **g)** -R2 cell surface expression in CIB1-depleted MDA-436 cells in relative to control cells at 2, 3, or 4 days post infection. Data represent means +/- SD (n=3). **h)** Representative DIC images (20x) of shControl (shCTRL), shCIB1-1, or shCIB1-2 MDA-436 TNBC cells. Insets show characteristic paraptotic morphology in CIB1-depleted cells (shCIB1) relative to control (shCTRL). **Please note that quantifications of cell death (Additional file 2: Figure S1B and S1D) and TRAIL-1/2 levels (Additional file 2: Figure S1F and S1G) using shCIB1-1 were taken from Figures 1, 2, 3, 4 solely to show side-by-side comparisons with shCIB1-2.

**Additional file 3: Figure S2.** CIB1 depletion plus docetaxel or TRAIL activates Bid and disrupts mitochondrial membrane potential. Mitochondrial apoptosis was further investigated by probing for a pro-apoptotic Bcl-2 related protein, Bid, and analyzing mitochondrial membrane potential by staining with JC-1. Control or CIB1-depleted MDA-436 cells were treated with docetaxel/TRAIL, followed by immunoblotting and JC-1 staining. Lysates from combination treatments involving **a)** docetaxel (n=2) and **b)** TRAIL (n=2) were probed for Bid and GAPDH (loading control using. **c)** Quantification of JC-1 aggregates (red) versus monomers (green) was used a surrogate for mitochondrial membrane potential. Data are represented in means +/- SD (n=3). p-value * <0.05; ** <0.01 compared to untreated control, two tailed t-test.

**Additional file 4: Figure S3.** CIB1 depletion plus docetaxel activates death receptor-mediated apoptosis in other TNBC cells. Caspase-8 activation is observed in TNBC cell lines treated with the combination of CIB1 depletion and the indicated concentrations of docetaxel. Control and CIB1-depleted **a)** MDA-468 (n=3) and **b)** MDA-231 (n=3) cells were treated with either vehicle (DMSO) or docetaxel as in Additional file 2: Figure S1B. Representative Western blot showing cleaved caspase-8 and GAPDH (lower panel, n=3).

**Additional file 5: Figure S4.** CIB1 depletion plus TRAIL increases death receptor-mediated apoptosis in a CIB1 depletion-sensitive TNBC cells. CIB1 depletion in combination with TRAIL induces cell death in CIB1-depletion sensitive but not insensitive TNBC cells. Control and CIB1-depleted **a)** MDA-468 and **b)** MDA-231 cells were treated with either vehicle (water) or TRAIL as in Additional file 2: Figure S1B. Percent cell death quantified as in Additional file 2: Figure S1 and is shown in means +/- SD (n=3) (*P < 0.05, **P < 0.01, ***P < 0.001, and ****P < 0.0001, ANOVA). Interestingly, increased caspase-8 activity in response to CIB1 depletion plus TRAIL was detected in both cells. Representative Western blots of 3 separate experiments showing PARP, cleaved caspase-8, CIB1, and GAPDH expression (lower panel).

**Additional file 6: Figure S5.** Combination of CIB1 depletion and docetaxel/TRAIL induces paraptosis. Paraptotic signaling was funder investigated by analyzing IGF-1R and JNK pathways. **a)** Control or CIB1 depleted MDA-436 cells were treated with either docetaxel (10 nM & 35 nM) or TRAIL (5 ng/mL & 10 ng/mL) as described in Figure 1. Lysates were probed for IGF-1R, phosphorylated JNK, total JNK, and GAPDH (n=2). **b)** To determine the contribution of paraptotic cell death, control or CIB1-depleted MDA-436 cells were pretreated with vehicle (DMSO) or 5 mM of the protein synthesis inhibitor cycloheximide for 24 h before adding 30 nM docetaxel or 10 ng/ml TRAIL for 48 h. Percent cell death was quantified and normalized to control, represented by means +/- SD (n = 3).

**Additional file 7: Figure S6.** CIB1 depletion may upregulate TRAIL-R1/R2 and IGF-1R expression in docetaxel-resistant TNBC cells. CIB1 depletion potentiates TRAIL-induced cell death in docetaxel-resistant MDA-436 cells potentially via upregulation of both TRAIL-R1 and –R2. **a)** Dose-response of docetaxel-induced cell death in parental (MDA-436-PR) versus docetaxel-resistant (MDA-436-DCX^R^) TNBC cells over 48 hr confirms resistance in MDA-436-DCX^R^ cells. Cell death was quantified using trypan blue exclusion assay. Data represents means +/- SD (n=2). FACS analysis of cell surface expression of **b)** TRAIL-R1 and **c)** TRAIL-R2 in CIB1 depleted (shCIB1) MDA-436-PR and MDA-436-DCX^R^ cells normalized to IgG-stained control cells (shCTRL) 4 days post infection with RNA interference. Data represent means +/- SD (n=3); * P < 0.05; ** P < 0.01. **d)** Representative Western blot from 3 separate experiments showing TRAIL-R1, TRAIL-R2, and vinculin (loading control) expression in MDA-436-PR and MDA-436-DCX^R^ cells 3 and 4 days post-infection with either shControl (Ctr) or shCIB1 (CIB1). **e)** Paraptotic signaling in a chemo-resistant setting was analyzed by probing for IGF-1R, phosphor-JNK, total JNK, and Rac (loading control) in control or CIB1-depleted parental and docetaxel-resistant TNBC cells treated with either docetaxel (10 nM) or TRAIL (5 ng/mL) for 48 h (n=2).

## Abbreviations

TNBC: triple-negative breast cancer
AKT: protein kinase B
ERK: extracellular regulated kinase
CIB1: calcium integrin-binding protein 1
TRAIL: tumor necrosis factor-related apoptosis-inducing ligand
EGFR: epidermal growth factor receptor
PI3K: phosphoinositide 3-kinase
MEK: mitogen-activated protein kinase
DR4/5: death receptor 4/5
Alix: ALG-2-interacting protein X
shRNA: short hairpin RNA
FACS: fluorescence-activated cell sorting
z-VAD-fmk: carbobenzoxy-valyl-alanyl-aspartyl-[*O*-methyl]-fluoromethylketone
LC-III: microtubule-associated proteins 1A/1B light chain 3B
p-RIPK1: phosphorylated receptor-interacting serine/threonine-protein kinase 1
c-FLIP: cellular FLICE-inhibitory protein
XIAP: X-linked inhibitor of apoptosis
IAP: inhibitor of apoptosis
GAPDH: glyceraldehyde 3-phosphate dehydrogenase
PARP: poly (ADP-ribose) polymerase
PBS: phosphate-buffered saline
JC-1: tetraethyl benzimidazolyl carbocyanine iodide
