# Supplementary figures and images for: CIB1 depletion with docetaxel or TRAIL enhances triple-negative breast cancer cell death
Source: Cancer Cell Int. 2019 Feb 4;19:26. doi: 10.1186/s12935-019-0740-2 (PMC6360800; doi:10.1186/s12935-019-0740-2)

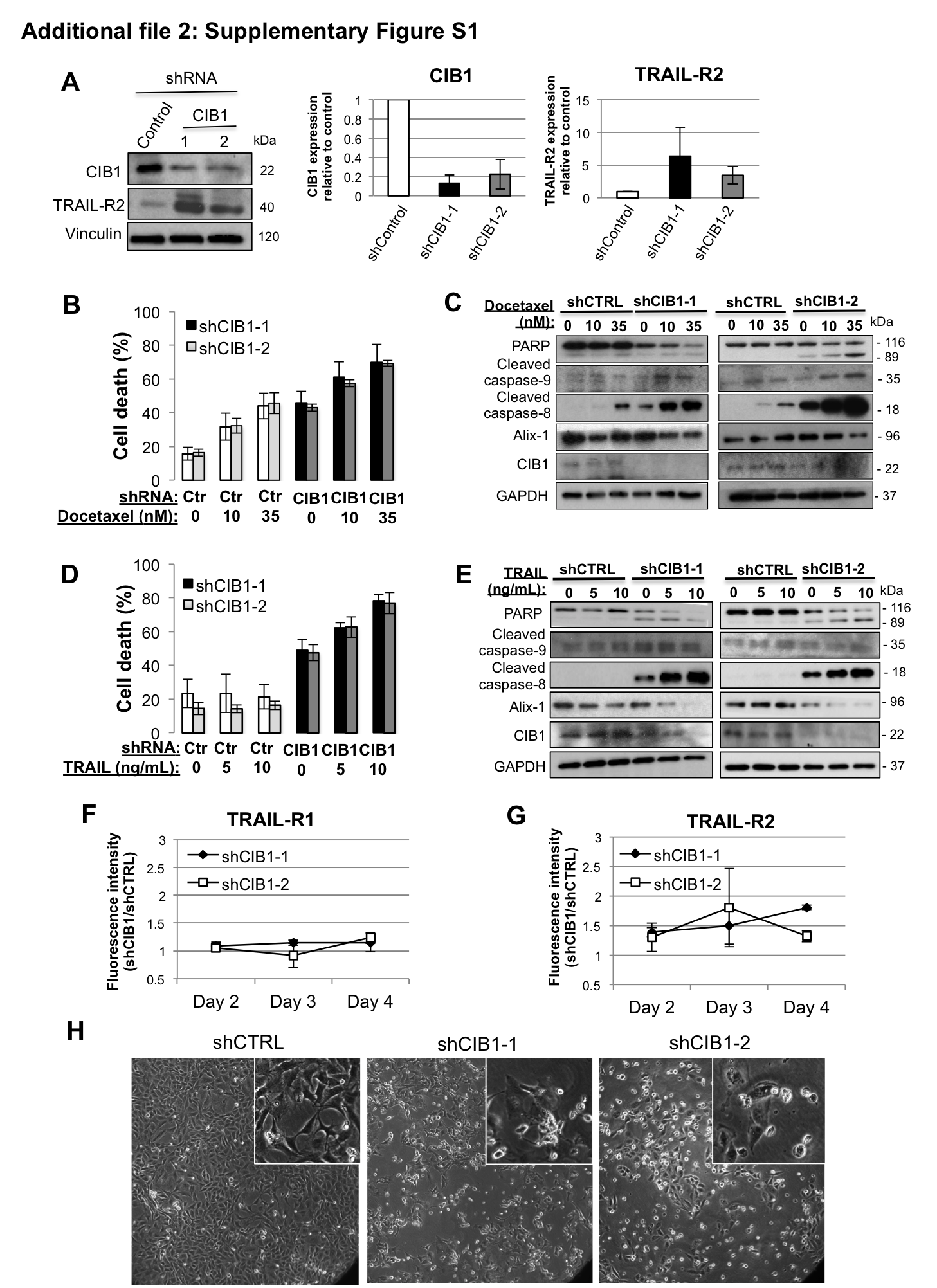

Supplement: Supplementary file 2 — Additional file 2: Figure S1. Validation of CIB1 depletion alone or in combination with docetaxel or TRAIL. MDA-436 TNBC cells were infected with either control or two different lentiviral CIB1 shRNA sequences (FG12 [1] and PLKO [2]). a) Western blot results followed by quantification via densitometry show similar CIB1 knockdown and TRAIL-R2 upregulation in MDA-436 cells infected with either CIB1 shRNA-1 or -2 for 96 h. Two days-post infection, cells were treated with vehicle control or, b) docetaxel (1 [n=5] and 2 [n=3]), or c) TRAIL (1 [n=3] and 2 [n=3]) for 48 h. Percent cell death was quantified via trypan blue exclusion assay and is shown as means +/- SD. Next, we examined death receptor-mediated apoptotic and paraptotic signaling induced by the combination treatment using CIB1 shRNA-1 or -2. Representative Western blot showing PARP, cleaved caspase-9, cleaved caspase-8, Alix, CIB1, and GAPDH in shControl (shCTRL) or shCIB1 (1 and 2) infected cells in combination with d) docetaxel (1 [n=5] and 2 [n=3]) or e) TRAIL (1 [n=3] and 2 [n=3]). FACS analysis of f) TRAIL-R1 and g) -R2 cell surface expression in CIB1-depleted MDA-436 cells in relative to control cells at 2, 3, or 4 days post infection. Data represent means +/- SD (n=3). h) Representative DIC images (20x) of shControl (shCTRL), shCIB1-1, or shCIB1-2 MDA-436 TNBC cells. Insets show characteristic paraptotic morphology in CIB1-depleted cells (shCIB1) relative to control (shCTRL). **Please note that quantifications of cell death (Additional file 2: Figure S1B and S1D) and TRAIL-1/2 levels (Additional file 2: Figure S1F and S1G) using shCIB1-1 were taken from Figures 1, 2, 3, 4 solely to show side-by-side comparisons with shCIB1-2. [file 12935_2019_740_MOESM2_ESM.tiff]

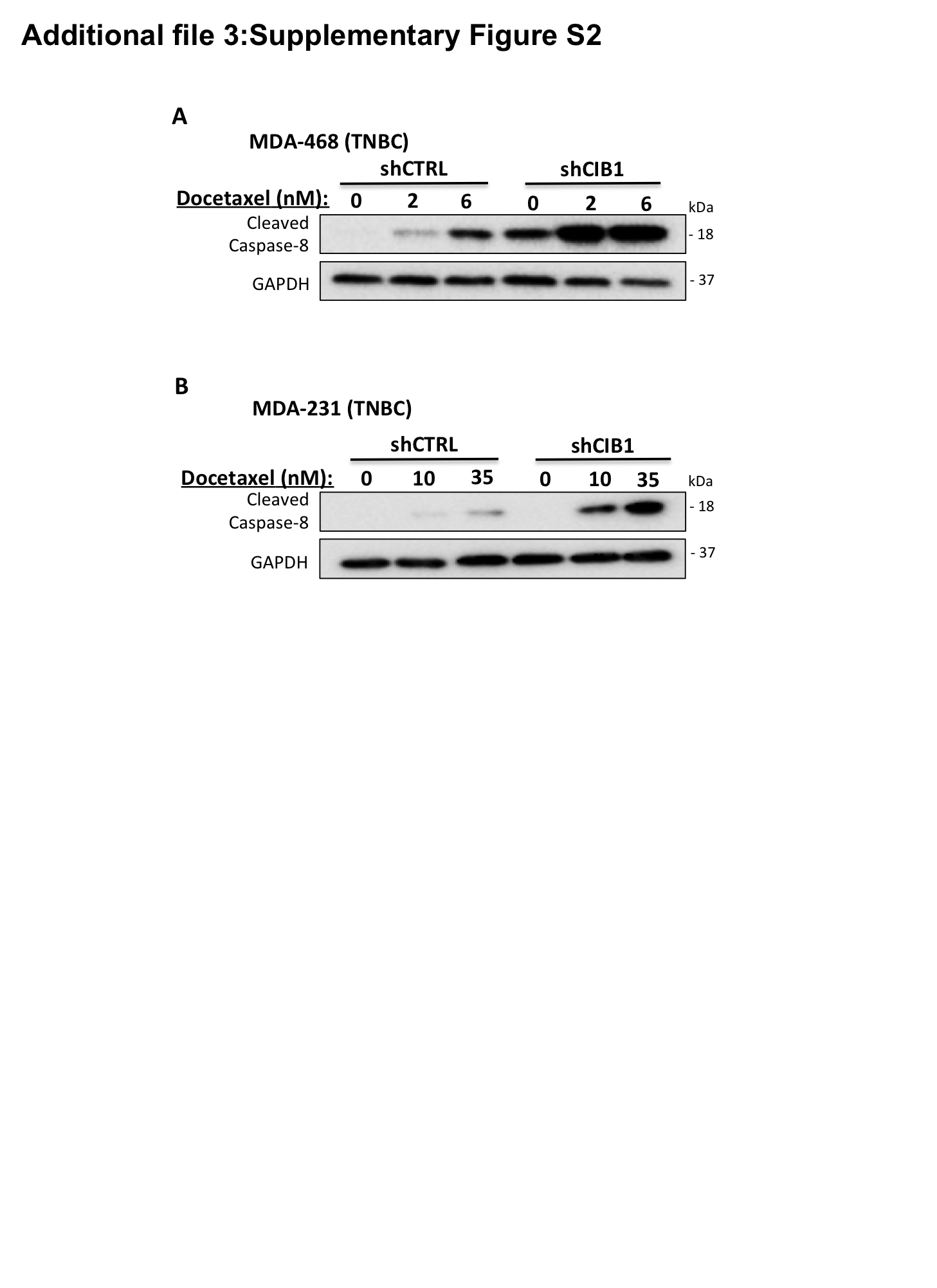

Supplement: Supplementary file 3 — Additional file 3: Figure S2. CIB1 depletion plus docetaxel or TRAIL activates Bid and disrupts mitochondrial membrane potential. Mitochondrial apoptosis was further investigated by probing for a pro-apoptotic Bcl-2 related protein, Bid, and analyzing mitochondrial membrane potential by staining with JC-1. Control or CIB1-depleted MDA-436 cells were treated with docetaxel/TRAIL, followed by immunoblotting and JC-1 staining. Lysates from combination treatments involving a) docetaxel (n=2) and b) TRAIL (n=2) were probed for Bid and GAPDH (loading control using. c) Quantification of JC-1 aggregates (red) versus monomers (green) was used a surrogate for mitochondrial membrane potential. Data are represented in means +/- SD (n=3). p-value * <0.05; ** <0.01 compared to untreated control, two tailed t-test. [file 12935_2019_740_MOESM3_ESM.tiff]

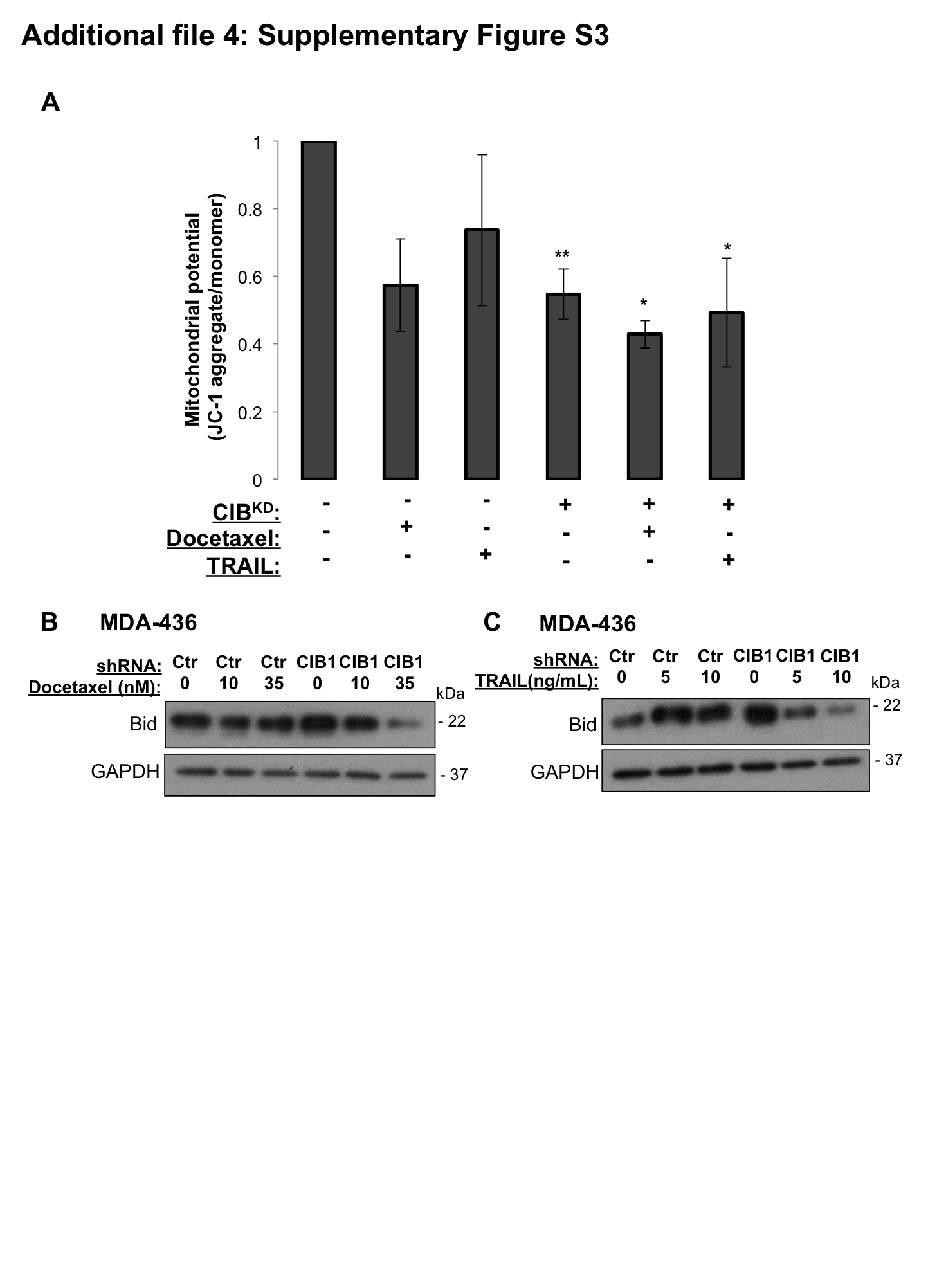

Supplement: Supplementary file 4 — Additional file 4: Figure S3. CIB1 depletion plus docetaxel activates death receptor-mediated apoptosis in other TNBC cells. Caspase-8 activation is observed in TNBC cell lines treated with the combination of CIB1 depletion and the indicated concentrations of docetaxel. Control and CIB1-depleted a) MDA-468 (n=3) and b) MDA-231 (n=3) cells were treated with either vehicle (DMSO) or docetaxel as in Additional file 2: Figure S1B. Representative Western blot showing cleaved caspase-8 and GAPDH (lower panel, n=3). [file 12935_2019_740_MOESM4_ESM.tiff]

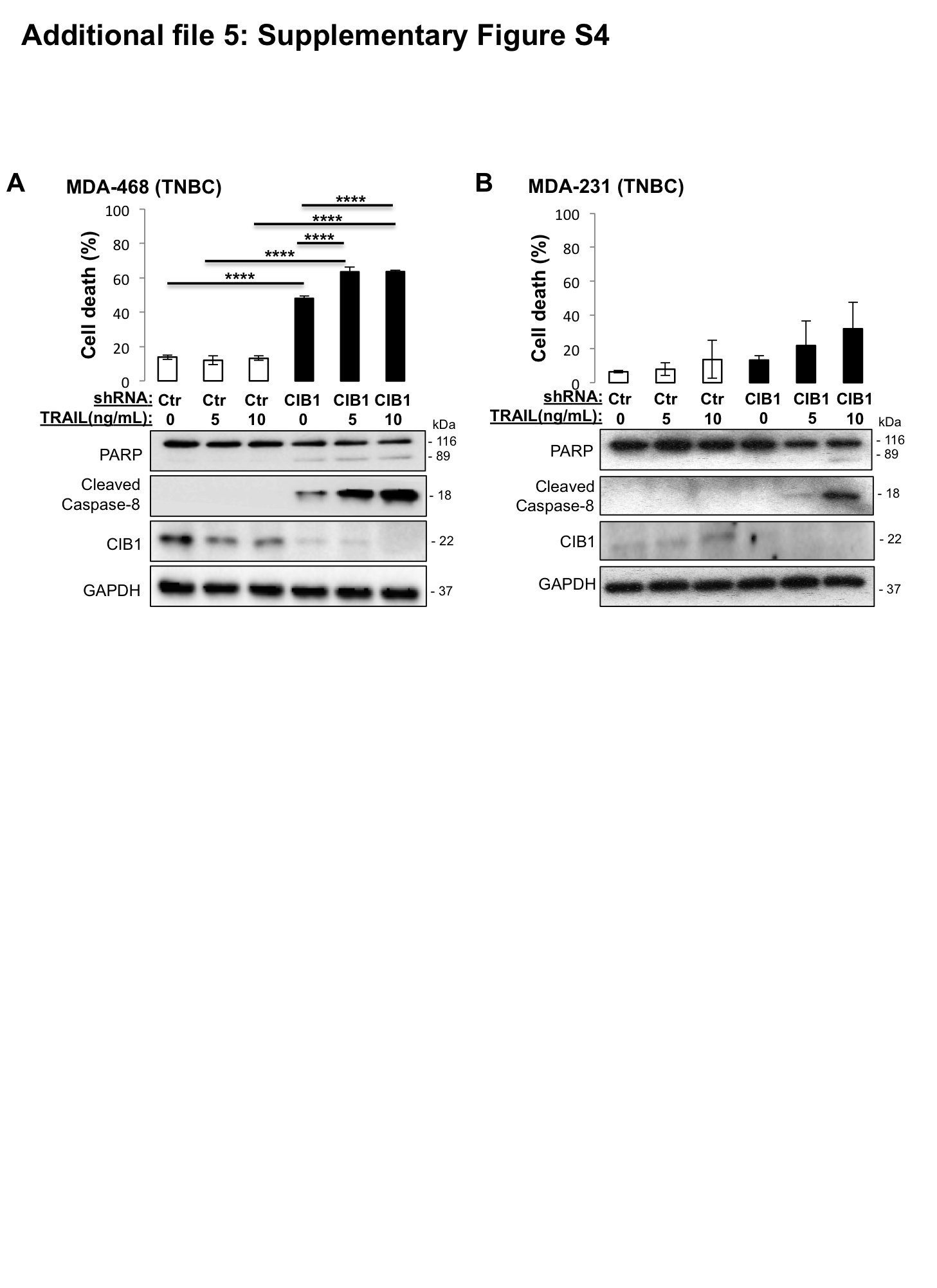

Supplement: Supplementary file 5 — Additional file 5: Figure S4. CIB1 depletion plus TRAIL increases death receptor-mediated apoptosis in a CIB1 depletion-sensitive TNBC cells. CIB1 depletion in combination with TRAIL induces cell death in CIB1-depletion sensitive but not insensitive TNBC cells. Control and CIB1-depleted a) MDA-468 and b) MDA-231 cells were treated with either vehicle (water) or TRAIL as in Additional file 2: Figure S1B. Percent cell death quantified as in Additional file 2: Figure S1 and is shown in means +/- SD (n=3) (*P < 0.05, **P < 0.01, ***P < 0.001, and ****P < 0.0001, ANOVA). Interestingly, increased caspase-8 activity in response to CIB1 depletion plus TRAIL was detected in both cells. Representative Western blots of 3 separate experiments showing PARP, cleaved caspase-8, CIB1, and GAPDH expression (lower panel). [file 12935_2019_740_MOESM5_ESM.tiff]

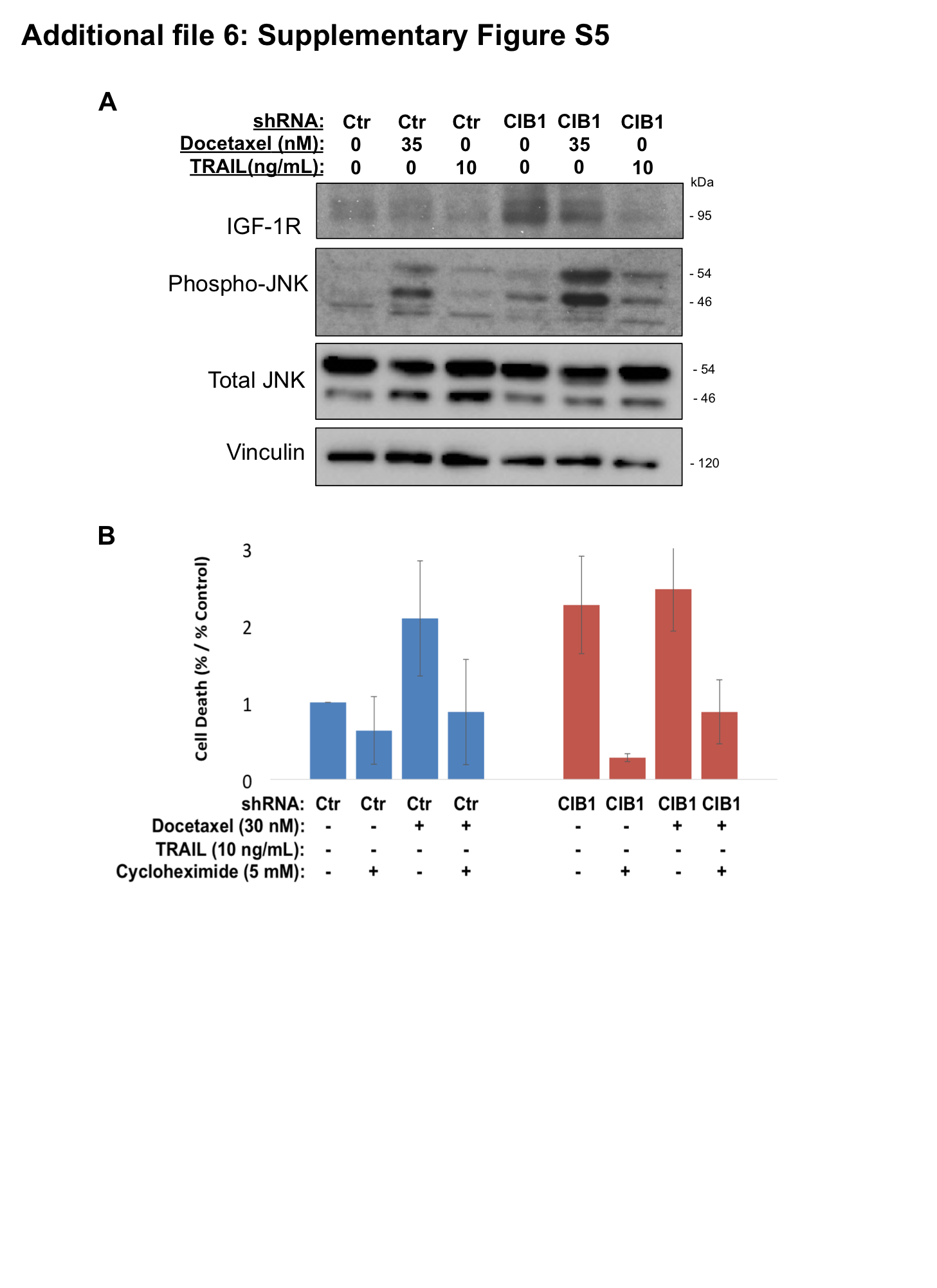

Supplement: Supplementary file 6 — Additional file 6: Figure S5. Combination of CIB1 depletion and docetaxel/TRAIL induces paraptosis. Paraptotic signaling was funder investigated by analyzing IGF-1R and JNK pathways. a) Control or CIB1 depleted MDA-436 cells were treated with either docetaxel (10 nM & 35 nM) or TRAIL (5 ng/mL & 10 ng/mL) as described in Figure 1. Lysates were probed for IGF-1R, phosphorylated JNK, total JNK, and GAPDH (n=2). b) To determine the contribution of paraptotic cell death, control or CIB1-depleted MDA-436 cells were pretreated with vehicle (DMSO) or 5 mM of the protein synthesis inhibitor cycloheximide for 24 h before adding 30 nM docetaxel or 10 ng/ml TRAIL for 48 h. Percent cell death was quantified and normalized to control, represented by means +/- SD (n = 3). [file 12935_2019_740_MOESM6_ESM.tiff]

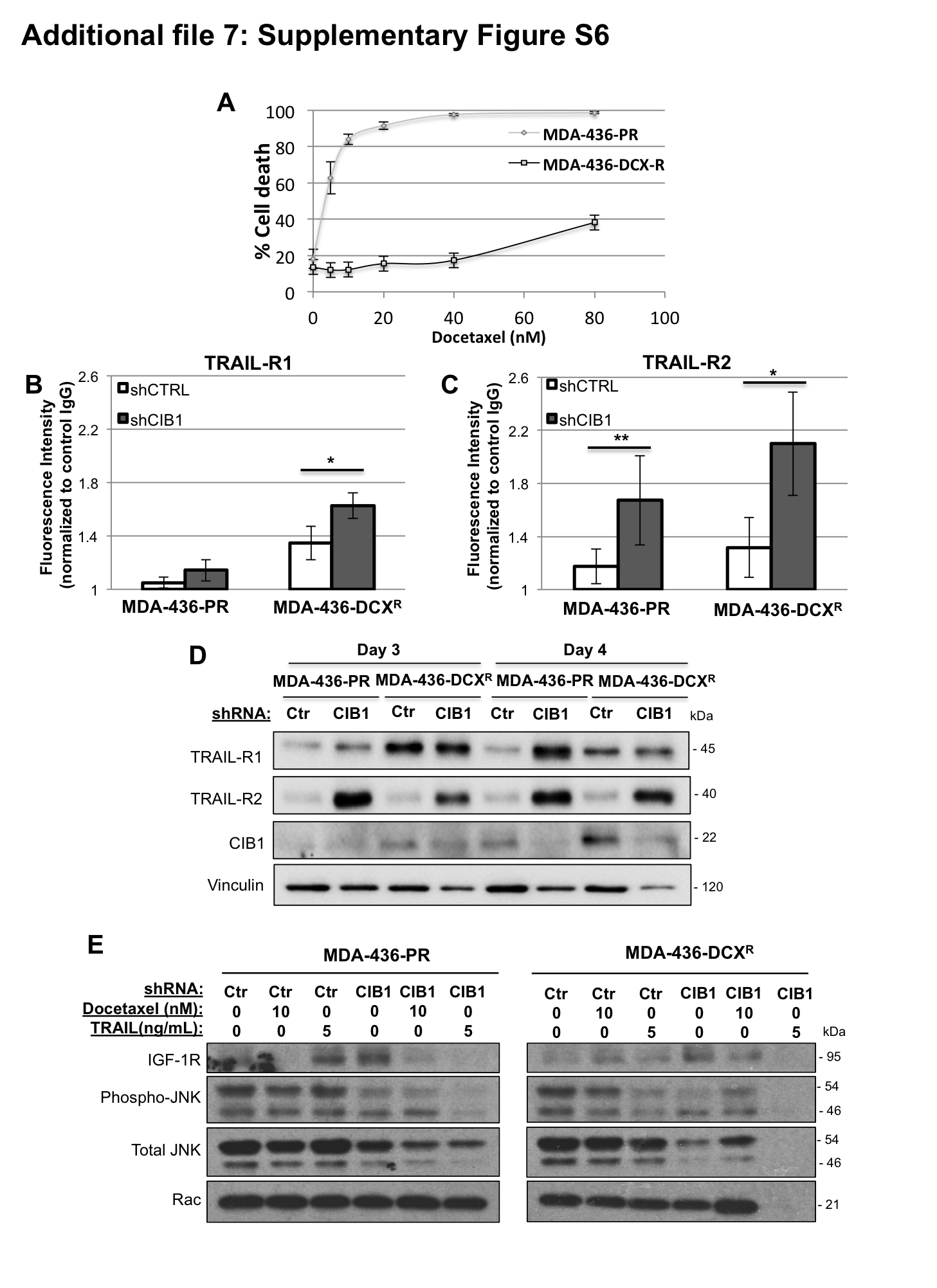

Supplement: Supplementary file 7 — Additional file 7: Figure S6. CIB1 depletion may upregulate TRAIL-R1/R2 and IGF-1R expression in docetaxel-resistant TNBC cells. CIB1 depletion potentiates TRAIL-induced cell death in docetaxel-resistant MDA-436 cells potentially via upregulation of both TRAIL-R1 and –R2. a) Dose-response of docetaxel-induced cell death in parental (MDA-436-PR) versus docetaxel-resistant (MDA-436-DCXR) TNBC cells over 48 hr confirms resistance in MDA-436-DCXR cells. Cell death was quantified using trypan blue exclusion assay. Data represents means +/- SD (n=2). FACS analysis of cell surface expression of b) TRAIL-R1 and c) TRAIL-R2 in CIB1 depleted (shCIB1) MDA-436-PR and MDA-436-DCXR cells normalized to IgG-stained control cells (shCTRL) 4 days post infection with RNA interference. Data represent means +/- SD (n=3); * P < 0.05; ** P < 0.01. d) Representative Western blot from 3 separate experiments showing TRAIL-R1, TRAIL-R2, and vinculin (loading control) expression in MDA-436-PR and MDA-436-DCXR cells 3 and 4 days post-infection with either shControl (Ctr) or shCIB1 (CIB1). e) Paraptotic signaling in a chemo-resistant setting was analyzed by probing for IGF-1R, phosphor-JNK, total JNK, and Rac (loading control) in control or CIB1-depleted parental and docetaxel-resistant TNBC cells treated with either docetaxel (10 nM) or TRAIL (5 ng/mL) for 48 h (n=2). [file 12935_2019_740_MOESM7_ESM.tiff]
